# Shading Net and Partial Covering Plastic Film Do Not Affect Phenology, Photosynthetic Activity or Fruit Quality Traits of Kensington Pride Mango

**DOI:** 10.3390/plants11243510

**Published:** 2022-12-14

**Authors:** Dario Scuderi, Giovanni Gugliuzza, Giuseppe Di Salvo, Federico Priola, Roberta Passafiume, Vittorio Farina

**Affiliations:** 1 Dipartimento di Scienze Agrarie, Alimentari e Forestali (SAAF)—Università degli Studi di Palermo, 90128 Palermo, Italy; 2 CREA—Research Centre for Plant Protection and Certification, SS 113 Km 245.500, 90011 Bagheria, Italy

**Keywords:** protected horticulture, *Mangifera indica* L., mediterranean climate, fruit color development, gas exchanges

## Abstract

Mango cultivation in a protected environment is becoming widespread in the Mediterranean basin where the species has to face unfavorable weather conditions which do not occur in its native cultivation areas. Besides open-air cultivation, greenhouses—and other protection systems such as shading nets and partial covering of plastic films—have been tested recently. In this study, we focused on assessing the effect of a shading net, and a partially covering plastic film, on the development of “Kensington Pride” mango fruit skin-color, its final quality, and the plants’ photosynthetic activity. A new method of measuring mango skin-color on different sides of the fruit is proposed. No difference was observed with regard to the observed parameters between the plants cultivated under the two different protection systems and those growing in the open air. It can, therefore, be stated that such cultivation techniques do not alter the development of the mango fruit and its appearance, nor the plant’s photosynthetic activity.

## 1. Introduction

The cultivation of mango (*Mangifera indica* L.) is becoming increasingly common in the Mediterranean basin [1,2], due to the European market’s increasing demand for tropical fruit, and also as a consequence of the ongoing climate change which is happening in the region [3,4]. As a species native to the tropical climate of the Indian peninsula, mango needs protection from extreme temperatures when grown in a temperate climate environment such as the Mediterranean [5,6,7]. In Sicily, growers usually protect mango trees, especially the young ones, with various techniques and materials such as windshields, be they natural or artificial; cultivation under full plastic greenhouses, also used for other tropical species such as papaya [8]; or other systems of protection from low temperatures. One example of the latter, which is common amongst growers, is bagging the canopy of the young mango trees during winter inside a non-woven fabric cover, which is placed on the leaves, and tied to the trunk, in order to protect leaves and buds from chilling damages [9]. This technique clearly presents the inconvenience of increasing the need for manual operations and labor required by the grower, resulting in a rise in cultivation costs. The cultivation under greenhouses, on the other hand, exposes the trees to the risk of heat stress due to the high temperatures that can be reached inside the greenhouse, which in the Mediterranean summers can reach values above 50 °C [10,11] and imply a high risk of damage to trees and fruits [12,13].

In the Mediterranean basin, mango is also particularly prone to a disease known as Bacterial Apical Necrosis (BAN), which is caused by the polyphagous bacterium *Pseudomonas syringae* pv. *syringae* [14,15,16]. The occurrence of the disease is increased after severe weather events such as hail, storms, and strong winds, which can cause wounds on leaves and buds, providing potential pathogen entrance sites [17,18,19].

Therefore, it is necessary to find solutions which give crops protection from extreme low temperatures and mechanical damage deriving from bad weather events whilst avoiding significant increases in daily temperatures during summer.

Systems of permanent protection of the orchards, which have a more hybrid nature, such as shading nets or partial plastic covers, could be the most efficient solution to meet the mango growers’ needs. Additionally, they can serve a double purpose, partially protecting the plants from cold temperatures and mechanical damage during winter [20,21] and reducing solar irradiation of fruits during summer to prevent sunburn [22,23,24]. Therefore, nets might be of great importance in the context of climate change, particularly in conditions of extreme events, such as in the case of hailstorms and heat waves. Netting systems indeed contribute to reducing plant vulnerability to the high irradiances far above the leaves’ light saturation point. [25]. However, they can affect tree physiology by inducing changes in the orchard’s microclimate on the basis of various factors such as net color, density, and shading degree [13,24,26]. Contrasting effects of various shade degrees on mango physiology are reported in the literature [27,28,29]. Several studies on different species have shown that shading increases the photosynthetic activity of plants to different levels, or, when this does not happen, it does not reduce it in any case [29,30,31,32,33,34,35]. Shading nets, on the other hand, have been observed to slow down the color development on the skin of fruits such as Cripps Pink, Royal Gala, and Fuji apples [23,36].

So, questions that might arise regarding the use of protection systems on mango are (i) does the protection modify the orchard environment to such an extent that the phenology and photosynthetic activity of the plants are affected? (ii) What are the effects of the protection system on the fruit’s aesthetic and internal quality?

In this study, we observed the development of mangoes growing in two different protection systems and compared them to those growing in the open air, from the moment of Full Flowering until harvest, whilst monitoring the relative environmental conditions. We followed the evolution of the fruit’s skin color and the photosynthetic activity of mangoes (cv. “Kensington Pride”) during the fruit development period and assessed the fruits’ quality at harvest.

## 2. Results

### 2.1. Environmental Conditions

#### 2.1.1. Temperatures

**Figure 1:**
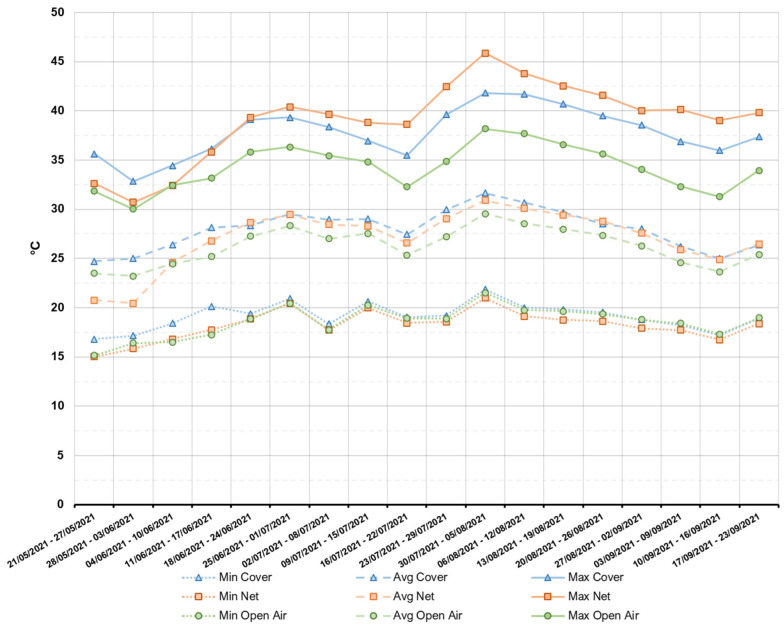
Weekly minimum, average, and maximum temperatures recorded in the three protection systems from the moment of Full Flowering until harvest of the fruits.

Data from the sensors placed in the plots corresponding to the three treatments (Figure 1) show that the protection systems had no significant impact on the minimum temperature. In fact, over the course of the observation period, the weekly minimum temperatures were similar in all three treatments. However, from the onset of summer (21 June), maximum temperatures in both Cover and Net were considerably higher than in the open air. The highest temperature, corresponding to 45.7 °C, was recorded in the Net treatment plot on 1 August.

#### 2.1.2. Light

**Figure 2:**
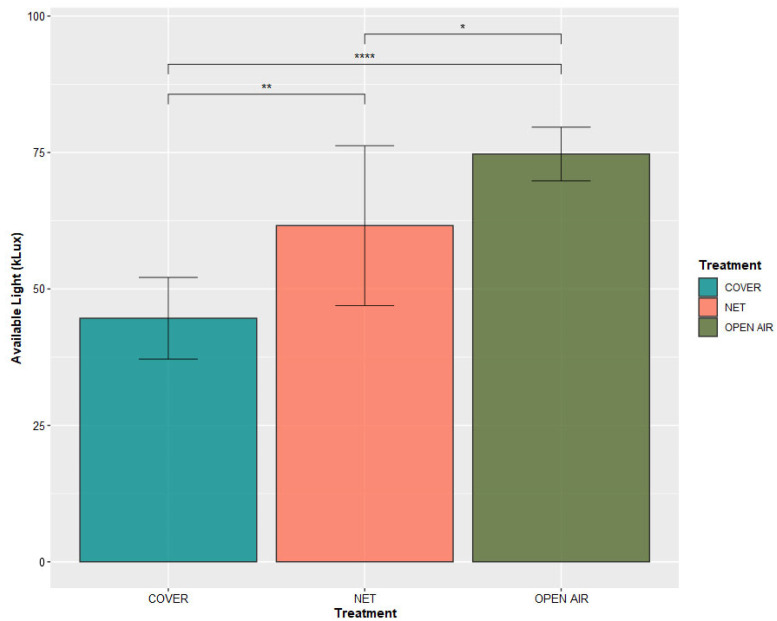
Values of kLux measured in the plots corresponding to each treatment. *, **, **** indicate statistically significant differences between any two treatments for Student’s *t*-test at *p*-value < 0.05, < 0.01, < 0.0001, respectively.

Significant differences were found in the quantity of light available for the plants of each treatment (Figure 2). The plants growing in the open air were receiving 74.7 ± 4.93 kLux, while for the plants in the Cover and Net treatments, this value was reduced by 40% and 20%, respectively.

### 2.2. Determination of the Date of Full Flowering

**Figure 3:**
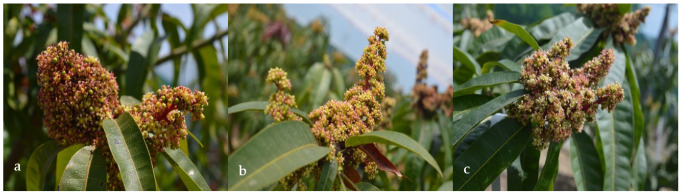
Panicles in the stage of Full Flowering photographed in the open air (**a**), Cover (**b**) and Net (**c**) treatments.

The shoots which did produce flowering, in all three treatments reached Full Flowering between 15 and 23 May 2022 (Figure 3). Therefore, the common date of 20 May was chosen as the average date of Full Flowering. The protection treatments did not cause any difference at the moment of reaching Full Flowering.

### 2.3. Photosynthetic Activity of Plants

**Figure 4:**
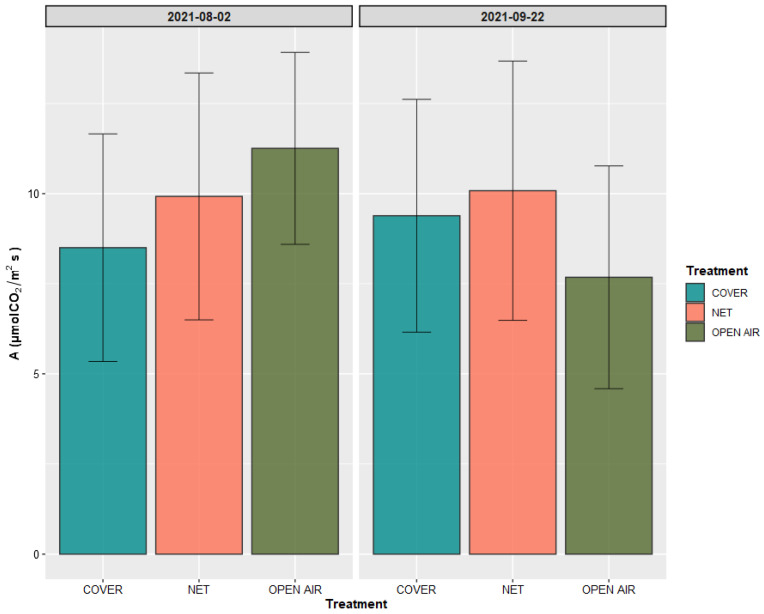
Net photosynthesis A measured on the plants of the three treatments in the two recording dates. Values are presented as mean ± standard deviation (*n* = 12).

Figure 4 shows the observed values of net photosynthesis of the plants for each treatment in the two measuring occasions.

The values of A were between 5 and 15 µmol CO_2_/m^2^ s in all samples of the three treatments. This is the range normally observed in mango trees [37]. Statistical analysis confirmed that there was no significant difference among the treatments on any of the measuring occasions. Plants growing in the open air showed the highest rates of gas exchange in the first measuring date at the beginning of August, whilst they showed a significant (*p*-value = 0.015) decrease in their A value on the second measuring date, which took place on the date of harvest. Plants growing in the two protection systems Cover and Net, instead, showed no significant difference in the values of A observed in the first and second measurement dates.

**Figure 5:**
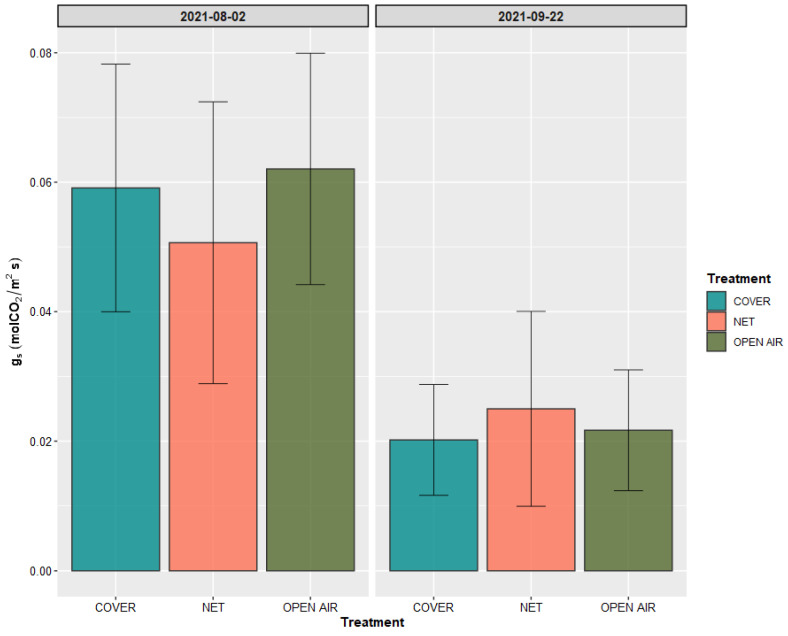
Stomatal conductance g_s_ measured on the plants of the three treatments in the two recording dates. Values are presented as mean ± standard deviation (*n* = 12).

Additionally, no difference was observed among the treatments with regard to the stomatal conductance g_s_ (Figure 5). However, all treatments showed a significant decrease in this value from the first to the second measuring date (*p*-value = 4.78 × 10^−5^, 6.00 × 10^−3^, 3.13 × 10^−6^ for Cover, Net, and open air, respectively).

### 2.4. Fruit Skin Color Evolution

**Figure 6:**
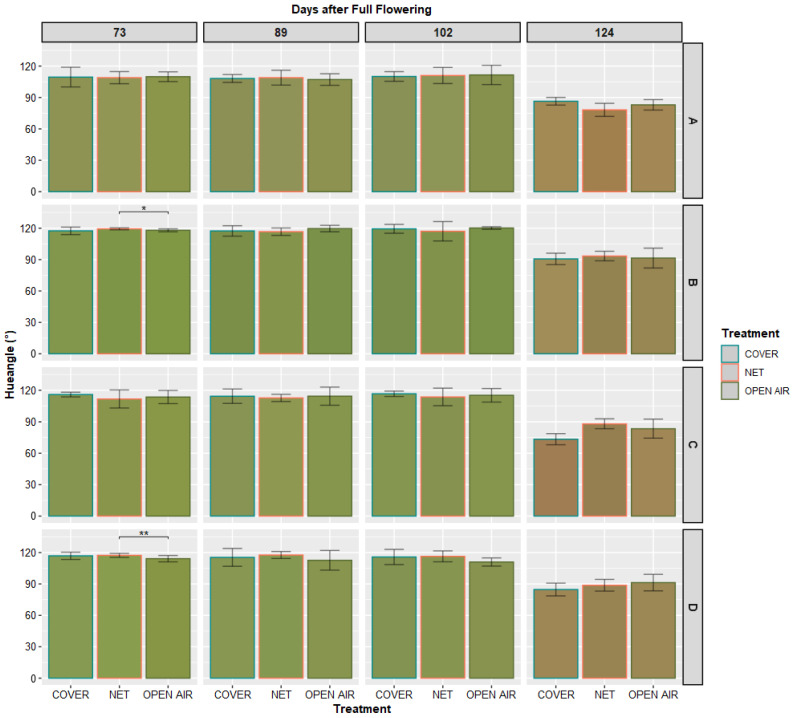
Mean values (*n* = 30) of hue angle (°) measured on the marked spots on the skin of the labeled fruits. The color of each bar corresponds to the average color of each spot (rows) at the given date (columns), transformed into visible RGB color space from the average values of CIE L*, a* and b* measured by the Chroma meter, using the R package colorspace [38]. The * and ** indicate significant differences between any two treatments for Student’s *t*-test at *p*-value < 0.05 and <0.01, respectively.

**Figure 7:**
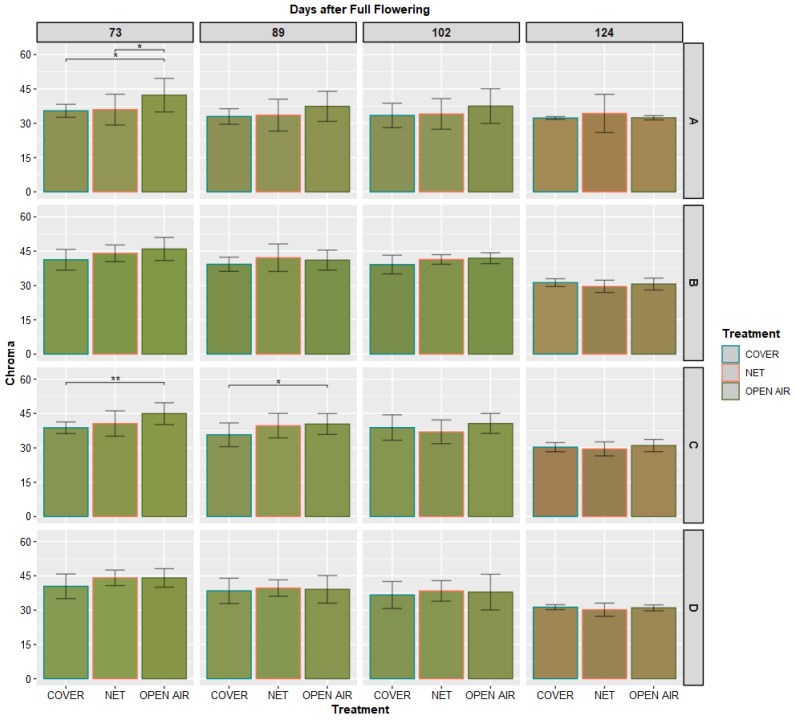
Mean values (*n* = 30) of Chroma (C*) measured on the marked spots on the skin of the labelled fruits. The color of each bar corresponds to the average color of each spot (rows) at the given date (columns), transformed into visible RGB color space from the average values of CIE L*, a* and b* measured by the Chroma meter, using the R package colorspace [38]. * and ** indicate significant differences between any two treatments for Student’s *t*-test at *p*-value < 0.05 and < 0.01, respectively.

Figure 6 and Figure 7 illustrate the average colors observed on each spot of the skin of the Kensington Pride fruits from when the first slight change in color was observed—73 days after Full Flowering—until harvest, which took place 124 days after Full Flowering. No significant difference could be observed among the treatments, after 90 days from Full Flowering.

Moreover, we could observe that the change in color from green to yellow/golden occurs only in the final stages of fruit development, which occur at 100 or more days after Full Flowering. Statistical analysis (Table 1), confirms that in the variety Kensington Pride, which is one of the mango varieties where skin color is most uniform over the whole fruit, there is no significant difference in the values of hue angle (*h*°) and Chroma measured in any part of the fruit until harvest.

Finally, the obtained average values of ΔE color difference (Table 2) confirm the absence of diversity in the final appearance of the fruits. Average values were between 1.28 and 5.24 at all measurement dates.

### 2.5. Fruit Quality Analyses

No sunburn damage was observed on any fruit following any of the treatments.

**Figure 8:**
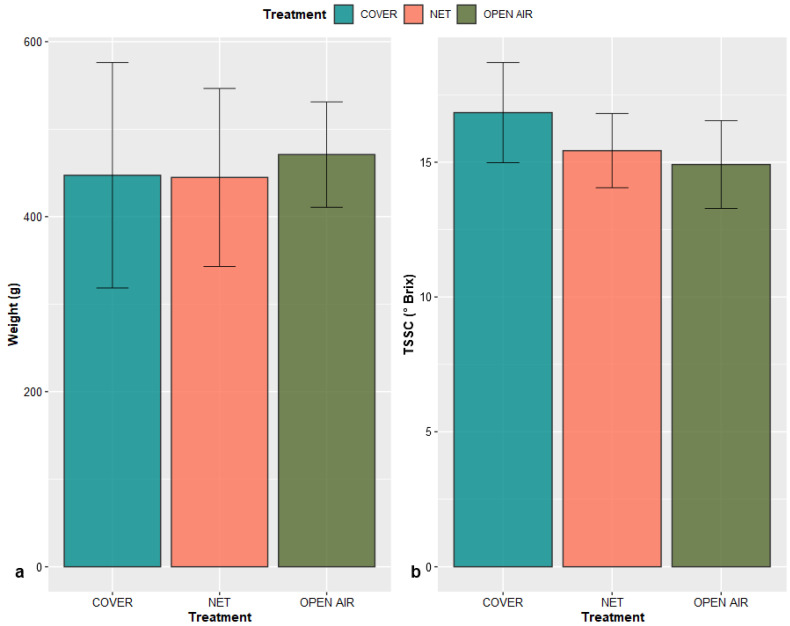
Weight (**a**) and Total Soluble Solids Content (**b**) of the fruits of the three different treatments measured at harvest. Values are presented as mean ± standard deviation of the harvested sample (*n* = 12) on each treatment.

The fruits of the three treatments showed no significant differences amongst their values of weight (*p*-value = 0.863), and Total Soluble Solids Content (*p*-value = 0.145). This confirms that the fruits were harvested at the mature-ripe stage, with a weight of around 0.5 kilos, and sugar content between 14 and 16.5 °Brix [39,40] (Figure 8). Even though the differences among the treatments were not found to be statistically significant, it can be observed that fruits in the two protection systems reached slightly higher TSSC values than the fruits growing in the open air. This reflects a greater sweetness of the fruit, which normally leads to a stronger appreciation by the consumer. This is probably a consequence of the higher average temperatures that were achieved during the fruit development period in both Cover and Net treatments. Factors which enhance fruit transpiration, in fact, are positively correlated with the content of sugars in the pulp of the fruit [41,42].

## 3. Discussion

**Figure 9:**
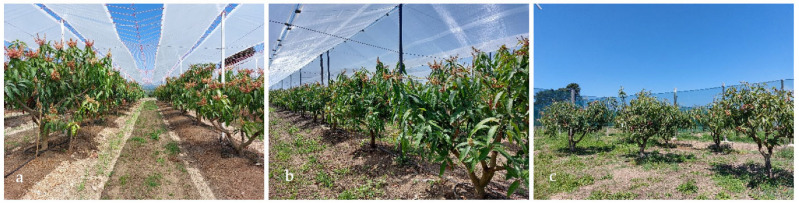
Views of the plots corresponding to the Cover (**a**), Net (**b**) and open-air (**c**) treatments.

Several studies available in the literature report contrasting effects of the presence of shading nets on the air temperature. Kurth et al., Alaphilippe et al., and Mira-Garcia et al. [43,44,45] all report lower temperatures recorded below the shade netting covering orchards of different crops, while Gimeno et al. and Blakey et al. [46,47] both found higher temperatures below the shade nettings compared to those in the open air. Such differing results are probably to be ascribed to microclimatic conditions and technical parameters, such as the color and shading intensity of the netting material, as well as its installation technique [13,48]. In our study, the highest temperatures were reached inside the Net rather than in the Cover treatment. This is probably due to the greater air circulation allowed by the latter compared to the Net cover system, which instead covers the “ceiling” and “walls” of the plot entirely (cfr. Figure 9).

It is also interesting to note that the dates of Full Flowering and harvest were not affected by the protection systems; this is due to their hybrid nature, which allows them to protect plants without the great shift in environmental conditions that are seen in traditional greenhouses [49,50]. This is of great importance to reduce the risk of exposing flowers to late frosts [51], and to allow the grower to easily plan routine cultural practices [52].

Fruit skin-color is an important feature of mango, with the fruit of many cultivars developing attractive pink to red coloration. Skin color is both environmentally and genetically determined, and typical of each variety [53]. Moreover, it is one of the factors which make the fruit more attractive to consumers [54]. The observed fruits were all uniformly colored, regardless of the protection treatment. An analogous result was also reported by Mthembu [55] on Kent mangoes. In the same study, the author also reports no difference in the TSSC measured in the fruits from different shading treatments.

The protection systems that we studied did not cause any significant difference in the photosynthetic activity of the Kensington Pride mango plants, in spite of different light availability conditions (Figure 2). We can assume that satisfactory conditions for the photosynthetic machinery of mango were satisfied in all three treatments studied, and that the process is not enhanced substantially with an increase in temperature or light availability after a certain threshold is reached [56,57,58]. The difference observed in the measured photosynthetic activity and stomatal conductance values between first and second recording occasions suggest a positive effect of the temperatures on the photosynthetic activity of mango trees in the open air. In fact, average temperatures in the Open Air equaled almost 30 °C in the week of the first measurement, while they had reduced to just above 25 °C on the second date (Figure 1).

Alternately, in the Net and Cover treatments, A values remained practically unaltered. We hypothesize that this happened because the maximum temperatures (Figure 1) recorded inside the protected plots in the period of the first measurement—5–10 °C higher than in the Open Air—limited the photosynthetic capacity of the trees [59]. In fact, Crafts-Brandner and Salvucci [60] report that net photosynthesis decreases, when temperatures exceed 35–40 °C, as was observed in both Net and Cover treatments, especially when paired with high CO_2_ concentrations.

However, more frequent measurements over a longer period should be taken before considerations of this kind are taken into account. We will limit ourselves to observe that, on two measurement occasions over the course of the fruit development period, the photosynthetic activity of the Kensington Pride mango trees was not affected, by the presence of a covering, consisting of either a shading net or a plastic film. This is in agreement with other studies, which compared the photosynthetic activity of the plants under shading nets, measured repeatedly with different climatic conditions [32,45] and has been observed several times in mango [61,62,63,64]. A significant effect of the shading nets on photosynthesis has been reported at various times in temperate species, notably apples [25,65,66,67].

## 4. Materials and Methods

### 4.1. Plant Material and Environmental Conditions

Five-year-old Mango (*Mangifera indica* L. cv. Kensington Pride) plants of comparable size were monitored in a commercial orchard at the Papamango farm located in Sant’Agata di Militello (Messina, Italy) (38°4′32″ N–14°39′1″ E). The first observations were made at Full Flowering, identified by the phenological stage 615 of the BBCH scale for mango [68], and continued until harvest. The climate of the area is identified as Mediterranean in the Koppen classification [69,70,71], with an average yearly temperature of 17–18 °C and average yearly rainfall between 800 and 1000 mm [7].

Plants were cultivated in the open air, and with two different covering systems: partial plastic covering (Cover), and Net covering (Figure 9).

Plants of the Open Air were used as control, as they had no cover above them, but were repaired by windshields on the sides, similar to the other treatments.

Plants of the Cover treatment were below a partial covering placed above the external, productive part of the canopy, leaving a small window in the central part of the plant, and a larger window between the rows. The cover was realized with a Non-Thermal Diffusive (NthD) plastic film (OROPLUS UV+, Plastik Advanced, Bergamo, Italy—thickness 160 μm, visible light transmission 80%, haze 30%), designed to protect the young shoots and fruits, from heavy rain damage and excessive solar radiation during the summer [72].

Plants of the Net treatment were below a continuous 30% shading of white plastic net installed in order to reduce sunburn damage on fruits, to achieve an increase in temperatures during winter, and to reduce wind speed and consequent mechanical damage in the orchard. All plants in the experiment were subjected to the same cultural practices and underwent the same management with regard to fertilization, irrigation, pruning, and pest-control treatments.

Temperatures in the plots corresponding to the three treatments were recorded with temperature-data loggers (Elitech RC-51, Elitech Ltd., London, UK) placed at the center of each plot after being properly calibrated. The difference in the quantity of light available for the trees in each plot was assessed using a lux meter (PCE LED-20, PCE Instruments, Meschede, Germany), at the same time, on the same day, within an interval of ten minutes, under clear sky conditions.

### 4.2. Field Measurements

#### 4.2.1. Determination of the Date of Full Flowering

Five trees per treatment were labeled. On each of them, three shoots per exposition were labeled and photographed at two-week intervals in order to identify the phenological stage according to the BBCH scale for mango [68,73]. Therefore, a total of 3 shoots × 4 expositions × 5 plants = 60 shoots per treatment were monitored to determine the date of Full Flowering. This moment was identified, as the date when a phenological stage between 610 and 619 was observed on more than 50% of the labeled shoots that had produced an inflorescence.

#### 4.2.2. Gas Exchanges Measures

Gas exchanges were measured twice using a LI-COR 6400 portable system (LI-COR, Lincoln, NE, USA). Data were recorded on three plants per treatment. Four readings per plant were carried out on four different leaves, each exposed to a different cardinal point (N-S-E-W). The measured parameters were photosynthetic activity A (µmol CO_2_/m^2^ s) and stomatal conductance g_s_ (mmol CO_2_/m^2^ s).

Measurements were carried out at 12:00 on both dates. On 2 August 2021, the conditions were: air temperature 35 °C, PAR (Cover 900; Net: 800, Open air 1300 μmol m^−2^ s^−1^); on 22 September 2021, the conditions were: air temperature 31 °C, PAR (Cover 800; Net: 750 Open air 1200 μmol m^−2^ s^−1^).

#### 4.2.3. Fruit Skin-Color Evolution

Mango skin-color is generally measured on harvested fruits [74,75]. The most detailed studies found in the literature only consider two or three points of measurement on the fruit’s surface [76,77,78]. In various mango cultivars, however, skin color is not uniform, and it develops at different moments of fruit development [79]. Typically, only the values of CIE coordinates L*, a* and b* are reported, which can be more difficult to compare than the values of Chroma and hue angle [80]. Here, we propose a new method to repeatedly measure skin color on the exact same spot throughout the fruit’s growth.

**Figure 10:**
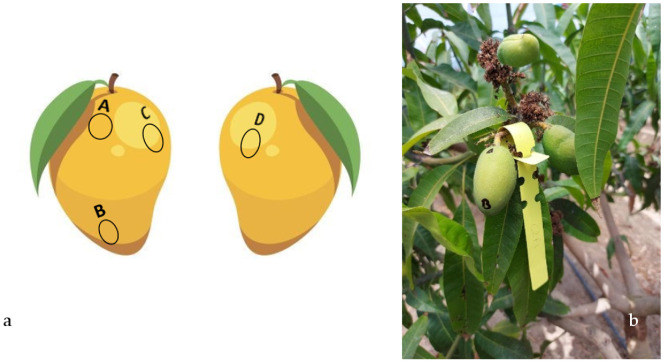
(**a**) Representation of the spots of the fruit surface on which skin color was measured. Spot A corresponded to the shoulder of the fruit most exposed to sunlight, spot B was just above the fruit apex, and spots C and D were on the two opposite cheeks. (**b**) One of the fruits observed during the study in the early stages of its growth.

After the fruit set, six fruits per plant, located all around the canopy, were labeled, and four different spots—A, B, C, and D (Figure 10a)—were marked on the surface of each labeled fruit, on five plants per treatment. The color of the fruit skin below each marked spot was recorded using a portable Konica Minolta CR-400 Chroma Meter (Konica Minolta Sensing Inc., Tokyo, Japan), starting at the first observation of color change, and at two-week intervals, until harvest. Values of L*, a* and b* read by the Chroma meter were then converted into hue angle (*h*°) and Chroma (C*) via Equations (1) [81]

(1) $$h{}^{\circ}=\left\{\begin{array}{c} \arctan\left(\frac{b^{*}}{a^{*}}\right)if\;a^{*}>0\;and\;b^{*}>0\\ 180+\arctan\left(\frac{b^{*}}{a^{*}}\right)if\;a^{*}<0\\ 360+\arctan\left(\frac{b^{*}}{a^{*}}\right)if\;a^{*}>0\;and\;b^{*}<0 \end{array}\right.$$

and (2) [82],

(2) $$C^{*}=\sqrt{a^{*2}+b^{*2}}$$

respectively.

Color difference [83] was then assessed between the protection systems and the open-air control using equation

(3) $$\Delta E=\sqrt{{\left(L_{o}-L_{t}\right)}^{2}+{\left(a_{o}-a_{t}\right)}^{2}+{\left(b_{o}-b_{t}\right)}^{2}}$$

where *x_o_* represents the parameter measured in the open air and *x_t_* is the parameter measured in any of the treatments.

### 4.3. Fruit Quality Analyses

Fruits were harvested on the same date, 124 days after Full Flowering, in all of the treatments, using external color and Total Soluble Solids Content (TSSC) of a sample of fruits as a harvesting index [79,84]. After harvest, 12 fruits from each treatment were transferred to a post-harvest laboratory, where they were subjected to the measurements of weight using a digital two-decimal precision scale (Gibertini, Italy), and total soluble solids content using a digital optical refractometer (Atago Co. Ltd., Tokyo, Japan). This was carried out in order to assess any difference in the degree of ripeness reached by the fruits for the three different treatments.

### 4.4. Statistical Analysis

Statistical analysis was performed using R software [85]. One-way ANOVA, at a significance level of *p*-value < 0.05, was conducted to assess the existence of differences among the treatments.

## 5. Conclusions

Protected horticulture systems are usually evaluated on the basis of their effectiveness in preventing damage to the crops during the cold season. However, in light of the rapid climate change and extreme climatic conditions that occur around the world with increasing frequency, it is necessary to observe the effect, and possible side effects, of protection systems on fruit species during all the different topical moments of their growing cycle.

We can confirm that shading nets and plastic film covers used in the hot dry summer of the Mediterranean climate do not have negative side effects on fruit skin-color development, or the phenology and photosynthetic activity of mango, during the reproductive cycle. Both the protection techniques used in this study had no significant effect on the observed parameters, when compared to the open-air control. Additionally, no significant difference could be observed between the shading net and the plastic film cover.

Obtaining a synchronous reproductive period, from flowering to harvest, and homogeneous fruit quality, regardless of the protection system, is a positive and interesting result in the optics of diffusion of such techniques.

More studies will certainly be needed to assess the effect of these protection systems during other critical periods of the mango life cycle in the Mediterranean climate.

## Figures and Tables

**Table 1:** F- and *p*-values of the analysis of variance of the effect of the Spot variable on hue angle (*h*°) and Chroma parameters at harvest, for each treatment.

			Hue Angle (*h*°)		Chroma	
Treatment	Effect	d.f.	F	*p*	F	*p*
COVER	Spot	3	1.46	0.262	1.55	0.241
NET	Spot	3	1.13	0.356	1.52	0.234
OPEN AIR	Spot	3	1.36	0.277	1.02	0.402

**Table 2:** Average values of color difference ΔE between the protection systems and the open-air-grown mango fruits at each measurement date.

	Days After Full Flowering			
ΔE	73	89	102	124 (Harvest)
COVER/OPEN AIR	5.24 ± 1.65	2.66 ± 1.81	2.53 ± 1.12	1.70 ± 1.45
NET/OPEN AIR	3.07 ± 0.65	1.28 ± 2.74	1.86 ± 0.75	2.57 ± 3.89

## Data Availability

The data presented in this study are available on request from the corresponding author. The data are not publicly available due to institution policy.
